# miR-126 Is Involved in Vascular Remodeling under Laminar Shear Stress

**DOI:** 10.1155/2015/497280

**Published:** 2015-06-28

**Authors:** Ana Mondadori dos Santos, Laurent Metzinger, Oualid Haddad, Eléonore M'baya-Moutoula, Fatiha Taïbi, Nathalie Charnaux, Ziad A. Massy, Hanna Hlawaty, Valérie Metzinger-Le Meuth

**Affiliations:** ^1^INSERM U1088, Faculty of Pharmacy and Medicine, University of Picardie Jules Verne, Rue des Louvels, 80037 Amiens, France; ^2^INSERM U1148, LVTS, UFR SMBH, University Paris 13 Sorbonne Paris Cité, 74 rue Marcel Cachin, 93000 Bobigny, France; ^3^Centre de Biologie Humaine (CBH), Amiens University Hospital, 80054 Amiens, France; ^4^Division of Nephrology, Ambroise Pare Hospital, Paris Ile de France Ouest (UVSQ) University, 09 avenue Charles de Gaulle, 92100 Boulogne Billancourt Cedex, France; ^5^University Paris 13 Sorbonne Paris Cité, UFR SMBH, 74 rue Marcel Cachin, 93017 Bobigny, France

## Abstract

Morphology and changes in gene expression of vascular endothelium are mainly due to shear stress and inflammation. Cell phenotype modulation has been clearly demonstrated to be controlled by small noncoding micro-RNAs (miRNAs). This study focused on the effect of laminar shear stress (LSS) on human endothelial cells (HUVECs), with an emphasis on the role of miRNA-126 (miR-126). Exposure of HUVECs *in vitro* to LSS modified the shape of HUVECs and concomitantly regulated the expression of miR-126, vascular cell adhesion molecule 1 (VCAM-1), and syndecan-4 (SDC-4). A significant upregulation of miR-126 during long-term exposure to flow was shown. Interestingly, LSS enhanced SDC-4 expression on the HUVEC membranes. Overexpression of miR-126 in HUVECs decreased the levels of targets stromal cell-derived factor-1 SDF-1/CXCL12 and VCAM-1 but increased the expression of RGS16, CXCR4, and SDC-4. No significant difference in terms of cell proliferation and apoptosis was observed between scramble, anti-miR-126, and pre-miR-126 transfected HUVECs. In Apo-E KO/CKD mice aortas expressing a high level of miR-126, SDC-4 was concomitantly increased. In conclusion, our results suggest that miR-126 (i) is overexpressed by long-term LSS, (ii) has a role in up- and downregulation of genes involved in atherosclerosis, and (iii) affects SDC-4 expression.

## 1. Introduction

Morphologic changes are observed in large vessels during the formation of atherosclerotic lesions, inducing substantial alterations in the phenotype of vascular wall cells, including endothelial and smooth muscle cells [1]. These changes recruit leukocytes that roll along the endothelial monolayer, thereby increasing the production of chemokines and cytokines. Chemokines are chemoattractant cytokines for leukocytes, including monocytes/macrophages, lymphocytes, and dendritic cells [2]. A chemokine such as SDF-1/CXCL12 is a CXC chemokine which binds to a family of specific G protein-coupled receptors (GPCRs) CXCR4 and plays an important role in hematopoiesis, development, and organization of the immune system [3]. SDF-1/CXCL12 induces the expression of adhesion molecules between endothelial cells and leukocytes, which transmigrate to the site of injury across the endothelial monolayer and initiate the inflammatory atherosclerotic process [4].

Vascular endothelial cells are submitted to various types of blood flow exerting tangential and perpendicular forces, the intensity of which plays an important role in endothelial cell homeostasis [5]. Straight parts of arteries are subjected to a unidirectional laminar shear stress (LSS). Atherosclerosis preferentially develops in arterial branches and curvatures, where the endothelial cell matrix layer comprising the cell surface heparan sulfates (HSs) is scarcely expressed. Syndecan (SDC) represents a large family of proteoglycans on the cell membrane and is the major source of heparan sulfate chains (HS). SDC use their HS chains to bind a variety of soluble and insoluble ligands, such as extracellular matrix components, growth factors, cytokines, and proteinases, all interacting with endothelial-specific receptors [6–8]. This interaction occurs either* via* direct binding of biomolecules to increase their local concentration or by blocking other biomolecules to prevent them from reaching the endothelial surface [9].

Modulation of cellular phenotypes is known to be controlled by a regulatory system of small noncoding micro-RNAs (miRNAs) with an approximate length of 22 nucleotides [10]. Mature miRNA binds by partial complementary base-pairing to mRNA and is therefore theoretically able to target a large number of mRNA, conferring a great versatility in the modulation of gene expression. Conversely, one mRNA can also be regulated by several miRNAs [11].

miR-126 is a strongly expressed microRNA specific to endothelial cells which fine-tunes their phenotype [12]. miR-126 expression is also affected in the course of several physiological and pathological processes, such as angiogenesis, atherosclerosis, and the proinflammatory process [13, 14]. Deletion of miR-126 causes loss of vascular integrity and produces defects in endothelial cell proliferation, migration, and angiogenesis [15].

We recently studied the expression of several miRNAs, including miR-126, in large vessels during various stages of chronic kidney disease (CKD) and atherosclerosis [16] and showed that miR-126 is increased in the aorta in murine models of these diseases. This miRNA is of special interest in the study of the endothelial phenotype, as it has been recently described that miR-126 directly targets both SDF-1/CXCL12 [14] and vascular cell adhesion molecule 1 (VCAM-1) [12]. On the other hand, Zernecke et al. [17] showed that the atheroprotective effects of human endothelial cell apoptotic bodies are mediated by miR-126, which inhibits the negative regulator RGS16, thereby enabling CXCR4 to stimulate enhanced expression of SDF-1/CXCL12 via ERK1/2.

The aim of the present study was (1) to observe the impact of laminar flow on human endothelial cell morphology and cytoskeleton distribution, (2) to analyze its effect on miR-126, adhesion molecule (VCAM-1), and syndecans (SDC-1, SDC-4), and (3) to study the miR-126 and SDC-4 levels in mice model of atherosclerosis with CKD. These pieces of information are important to determine the altered interactions between monocytes in the blood and endothelial cells of the blood-vessels in early phase of atherosclerosis. We show that morphologic and genetic changes due to LSS induce the production of endogenous miR-126, which in turn affects the regulation of chemokines and proteoglycans.

## 2. Materials and Methods

### 2.1. Cell Culture

Human umbilical vein endothelial cells (HUVEC, CRL-1730, ATCC) were grown in ECBM culture medium (Endothelial Cell Basal Medium, PromoCell) supplemented with 12% fetal bovine serum (FBS), 5 ng/mL EGF (Epidermal Growth Factor), 0.2 mg/mL hydrocortisone, 0.5 ng/mL Vascular Endothelial Growth Factor (VEGF), 10 ng/mL basic Fibroblast Growth Factor (bFGF), 20 ng/mL R3 Insulin-like Growth Factor (IGF-1), 1 g/mL ascorbic acid, 1% penicillin-streptomycin (Invitrogen), and 1% L-glutamine (Invitrogen). The medium was changed twice a week. For the various experiments, the cells were maintained in either plastic T25-plates (25 cm^2^, 1.2 × 10^6^ cells/plate), 6-well glass plates (Labtek) (5 × 10^3^/well), 96-well plastic plates (5 × 10^3^ cells/well), 6-well plastic plates (2 × 10^5^ cells/well), or Ibidi *μ*-Slides (I 0.4 Luer, ibiTreat) (3 × 10^5^ cells/slide) under controlled humid atmosphere at 37°C with 5% CO_2_.

### 2.2. Laminar Shear Stress

To analyze the influence of shear stress on cell shape, LSS studies were performed on HUVECs. Cells were seeded in Ibidi slides (*μ*-Slide I 0.4 Luer, Ibidi, Biovalley, France). After 16 h of treatment, cells were submitted to flow conditions in complete culture medium with a shear stress at 0.8 dyne/cm^2^ (speed 0.6 mL/min) and two different flow times, 10 min and 24 h. For the 10 min flow time, the Ibidi slides were connected to the syringe pump system. For the long flow time (24 h), the Ibidi slides were connected to a closed-loop perfusion system with a peristaltic pump and maintained at 37°C with 5% CO_2_.

### 2.3. Immunolabeling

To identify the presence of SDC-1 and SDC-4 proteoglycans on the surface of human endothelial cells at the end of LSS, cells were fixed with 1% paraformaldehyde (PFA) for 30 min at 4°C. Cells were incubated for 1 h at 4°C with primary antibodies directed against SDC-1 (10 *μ*g/mL, monoclonal mouse IgG1, DL-101 Santa Cruz, TEBU, Biotechnology, Inc.), SDC-4 (10 *μ*g/mL, polyclonal rabbit IgG H-140, Santa Cruz, TEBU, Biotechnology, Inc.), and VCAM-1 (10 *μ*g/mL, Rat monoclonal IgG1 M/K-2, Santa Cruz,) or their isotypes. Anti-SDC-1 and anti-SDC-4 immunolabeling were revealed by Alexa Fluor 555 goat anti-mouse (IgG (*γ*2b)) or Alexa Fluor 555 goat anti-rabbit (IgG (H+L)) and Alexa Fluor 488 goat anti-rat (IgG (H+L)), respectively (both 1/100, Molecular Probes, Invitrogen, Cergy-Pontoise, France). All samples were also incubated with 1 mg/mL 4,6-diamidino-2-phenylindole hydrochloride (DAPI) solution (Sigma-Aldrich). Representative immunohistochemistry photomicrographs were taken using a Zeiss Axiophot microscope (Zeiss, AXIOPHOT, MicMac, Le Pecq, France) and fluorescence intensity was analyzed with IMAGE J software.

### 2.4. qRT-PCR

Some HUVECs were submitted to LSS for 10 min and 24 h. RNAs were then isolated with the mirVana miRNA Isolation Kit (Applied Biosystems) according to the manufacturer's instruction. DNAse was used to digest DNA, and reverse transcription was performed using High Capacity cDNA Synthesis Kit (Applied Biosystems). PCR reactions were performed with TaqMan Universal Master Mix (Applied Biosystems) and the following target probes (Applied Biosystems): hsa-miR-126 (UCGUACCGUGAGUAAUAAUGCG). Hs.00896423_ml (SDC-1), Hs.01120909_ml (SDC-4), Hs.00607978_s1 (CXCR4), and Hs.413297 (RGS16), hs.00171022_ml (SDF-1/CXCL12), Hs.107740 (Krüppel-like Factor, KLF-2), and Hs.109225 (VCAM-1) were measured using Power SYBR Green Mix (Bio-Rad) according to the manufacturer's instruction. The U6 small nuclear RNA (for miRNAs) and GAPDH (for mRNA targets) were used as endogenous controls.

### 2.5. miR-126 Transfection

HUVECs were detached, quantified, and transfected in suspension with anti-miR-126 or pre-miR-126 or control scramble in the presence of siPort NeoFX Transfection agent according to the manufacturer's instruction (Ambion). Transfected cells were seeded in 6-well plastic plates (2 × 10^5^/well) and cultured for 48 h at 37°C with 5% CO_2_. Cells then were trypsinized, and RNAs were isolated and stored at −80°C for further analysis by qRT-PCR.

### 2.6. MTT Assay

HUVECs cell metabolic activity was measured by reduction of MTT (Sigma-Aldrich). Cells (5 × 10^3^/well) were transfected with anti-miR-126 or pre-miR-126 or control scramble for 48 h, as previously described [18]. Cells were then incubated with 0.5 mg/mL MTT for 1 h at 37°C. After MTT withdrawal, the resulting blue formazan crystals were solubilized in DMSO (Merck, Fontenay-sous-Bois, France) and absorbance was determined at 595 nm.

### 2.7. Flow Cytometry Analysis

The Annexin V-FITC ApoptosisDetection Kit(BD Biosciences, USA) was used to verify the apoptosis level of HUVECs transfected by pre-miRNA.HUVECs were transfected with anti-miR-126 or pre-miR-126 or control scramble for 48 h, as previously described. Cells were then incubated with Annexin V-FITC for 30 min according to the manufacturer's instruction and flow cytometry was performed with a Becton-Dickinson FACS Calibur flow cytometer.

### 2.8. Animals: Diet and Surgical Procedures

All animal studies complied with the principles of Directive 2010/63/EU of the European Parliament and all protocols were approved by our Institution's Animal Care and Use Committee (*Comité Régional d'Ethique en Matière d'Expérimentation Animale de Picardie*, CREMEAP). The experiments were performed in female C57/BL6 mice purchased from Charles Rivers (Lyon, France). Animals were housed in polycarbonate cages in temperature- and humidity-controlled rooms with a 12:12-hour light-dark cycle and were given standard chow (Harlan Teklad Global Diet 2016, Harlan, Oxon, UK) and tap water* ad libitum*. Chow composition was 4.2% (wt/wt) fat, 16.7% protein, 60.89% carbohydrates, 0.98% calcium, 0.25% sodium, and 0.65% phosphorus. Mice were anesthetized with ketamine and xylazine (80 mg/kg and 8 mg/kg, resp.), and every effort was made to minimize suffering. At 8 weeks of age, mice were assigned to the following groups: wild-type (WT) mice which were submitted to sham operations (WT SHAM); WT mice with chronic kidney disease (WT CKD); Apolipoprotein-E knockout (Apo-E KO) mice which were submitted to sham operations (Apo-E KO SHAM); and Apolipoprotein-E knockout mice with CKD (Apo-E KO CKD). CKD was induced by applying cortical electrocautery to the right kidney and left total nephrectomy was then performed 2 weeks after the first operation [16]. After 10 weeks of uremia, animals were sacrificed and the whole aorta was removed surgically and stored at −80°C until further use.

### 2.9. Statistical Analysis

Data are expressed as mean ± S.D. Statistical comparisons were performed globally by one-way ANOVA and between two groups with a two-tailed Student's *t*-test. A *P* value <0.05 was considered significant (^*∗*^ *P* < 0.05, ^*∗∗*^ *P* < 0.01, and ^*∗∗∗*^ *P* < 0.0001).

## 3. Results

### 3.1. LSS Modified Morphology and F-Actin Distribution in HUVECs

**Figure 1:**
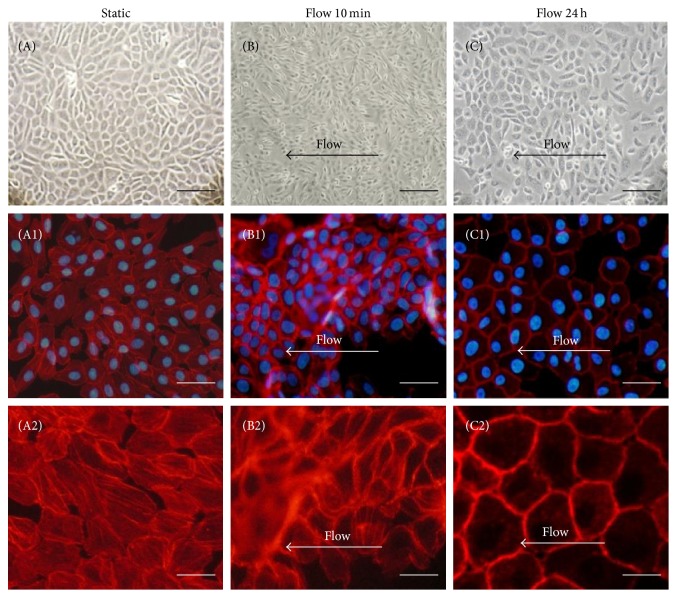
LSS alters the morphology of HUVEC cells. Representative phase contrast (A–C) or fluorescence (A1–C2) photographs of endothelial cells. HUVECs were grown under static conditions (A–A2) or LSS conditions for 10 min (B–B2) or 24 h (C–C2). Cells were then stained with Phalloidin (F-actin: red) and DAPI (Nucleus: blue). Global distribution of F-actin and cellular shape was analyzed for static conditions (A1), 10 min of LSS (B1) or 24 h of LSS (C1). High resolution zoom images of F-actin distribution in endothelial cells (A2-C2). Direction of flow is indicated by white arrows. For A–C scale bar = 40 *μ*m, for A1–C1 scale bar = 20 *μ*m, and for A2–C2 scale bar = 5 *μ*m.

LSS has been described to be a factor that does not cause damage to the vessel wall. However, altered morphology of endothelial cells exposed to LSS has only been described after long-term exposure [19]. In order to validate LSS in this endothelial cell model, HUVECs were exposed to three different durations of laminar flow culturein the Ibidi flow chamber: 10 min and 24 h with the same physiological wall shear stress at 0.8 dyne/cm^2^ [20]. The first step of our experiment was to optimize the cell quantity at day 1 of cell culture in IBIDI chamber to obtain a homogeneous endothelial cell monolayer without excessive cell condensation. The second step was to optimize the shear stress (dyne/cm^2^) which has been applied during all time of experiment, in our study for 10 min and 24 h, without any degradation of cell monolayer (data not shown). We decided to use IBIDI chambers without any additional layer on it (fibronectin, or collagen), and the highest shear stress (flow velocity) obtained without any cell detachment for 24 hours was 0.8 dyne/cm^2^ which was used throughout the paper. Before LSS, (under static-control conditions), HUVECs presented physiological morphology with a homogenous F-actin distribution throughout the cytoplasm. No changes in F-actin distribution and cell shape were observed after 10 min of LSS. Interestingly, after 24 h of LSS, the cells presented a cobblestone appearance with F-actin distribution confined to the membrane (Figure 1).

### 3.2. Long-Term LSS Increased Syndecan-1 and Syndecan-4 but Not VCAM-1 Expression in HUVECs

**Figure 2:**
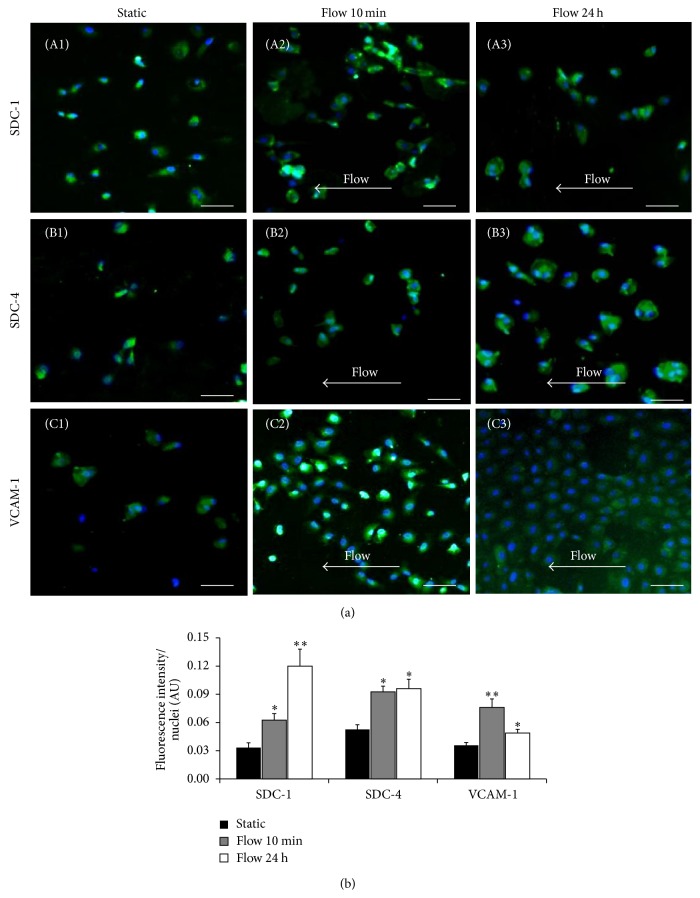
LSS induces SDC-1, SDC-4, and VCAM-1 expression in HUVECs. (a) Representative immunofluorescence staining of SDC-1, SDC-4, and VCAM-1 protein on endothelial cells cultured under static or LSS conditions for 10 min and 24 h. HUVEC cells were immunolabeled with anti-SDC-1 (A1–3), anti-SDC-4 (B1–3), and anti-VCAM-1 (C1–3) antibodies (green) or with their respective control isotypes. The nuclei were stained with DAPI (blue). High resolution zoom images of cells were added to each figure. Scale bar = 20 *μ*m. (b) Immunofluorescence quantification of proteins normalized with the nuclei number (*n* = 3) was done and 5 different fields in HUVECs submitted to static conditions and LSS (10 min and 24 h) were counted and presented as fluorescence intensity/nuclei (AU: arbitrary units). A progressive increase of SDC-1 and SDC-4 expression up to 24 h and an increase at 10 min and then a decrease of VCAM-1 expression were observed. One-way Global ANOVA and Student's *t*-test, for SDC-1: ^*∗*^ *P* < 0.05 (10 min of LSS versus static), ^*∗∗*^ *P* < 0.001 (24 h of LSS versus static); for SDC-4: ^*∗*^ *P* < 0.05 (10 min of LSS versus static), ^*∗*^ *P* < 0.05 (24 h of LSS versus static); for VCAM-1: ^*∗∗*^ *P* < 0.001 (10 min of LSS versus static), ^*∗*^ *P* < 0.05 (24 h of LSS versus static).

Syndecan-1, syndecan-4 (SDC-1 and SDC-4), and VCAM-1 expressions were analyzed at the beginning of the experience, under static condition, and after 10 min and 24 h of LSS (Figures 2(a) and 2(b)). Then, HUVECs were incubated with anti-SDC-1, anti-SDC-4, and anti-VCAM-1 antibodies to study its membrane expression by fluorescent microscopy. The total fluorescence intensity was normalized by cell number in each field. The results are presented in arbitrary units (AU)/nuclei and shown in Figure 2(b) There was a significant increase of SDC-1 (0.03 ± 0.005 versus 0.06 ± 0.007), SDC-4 (0.05 ± 0.005 versus 0.09 ± 0.006), and VCAM-1 (0.04 ± 0.003 versus 0.08 ± 0.009) expression observed after 10 min of LSS, as compared to static condition (Figure 2(b)). In addition, a significant increase of SDC-1 (0.03 ± 0.005 versus 0.12 ± 0.018), SDC-4 (0.05 ± 0.005 versus 0.096 ± 0.01), and VCAM-1 (0.04 ± 0.003 versus 0.049 ± 0.004) expression was observed after 24 h of LSS, as compared to static conditions (Figure 2(b), *P* < 0.05). Interestingly, only SDC-1 level was significantly increased by 1.8-fold (0.06 ± 0.007 versus 0.12 ± 0.018) between 10 min and 24 h of LSS. There were no changes of SDC-4 level (0.09 ± 0.006 versus 0.096 ± 0.001) and there was a 1.5-fold decrease of VCAM-1 level (0.08 ± 0.009 versus 0.049 ± 0.004) between 10 min and 24 h of LSS (Figure 2(b)). For these analyses we used the ANOVA statistic tests using Stat View software (^*∗*^ *P* < 0.05, ^*∗∗*^ *P* < 0.001). Interestingly, only SDC-1 level was significantly increased by 1.8-fold (0.06 versus 0.12) between 10 min and 24 h of LSS. There were no changes of SDC-4 level (0.09 versus 0.096) and there was a 1.5-fold decrease of VCAM-1 level (0.08 versus 0.049) between 10 min and 24 h of LSS (Figure 2(b)). For these analyses we used the ANOVA/ANCOVA statistic tests using Stat View software (^*∗*^ *P* < 0.05, ^*∗∗*^ *P* < 0.001).

### 3.3. LSS Modulated KLF-2 Level in HUVECs

**Figure 3:**
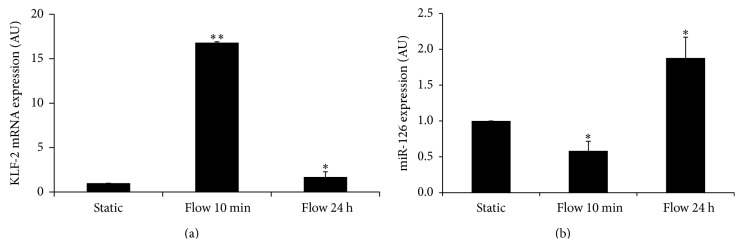
LSS increases KLF-2 mRNA expression and regulates miRNA expression in HUVECs. HUVECs cells were exposed to various durations of laminar shear stress (10 min and 24 h). (a) LSS increased KLF-2 mRNA expression. Values are expressed as mean ± SD of 3 experiments. (b) 24 h of LSS increased miR-126 levels in HUVECs. Values are expressed as mean ± SD of 3 independent experiments (AU: arbitrary units). Student's *t*-test, for KLF-2: ^*∗∗*^ *P* < 0.001 (10 min of LSS versus static), ^*∗*^ *P* < 0.05 (24 h of LSS versus static); for miR-126: ^*∗*^ *P* < 0.05 (10 min of LSS versus static), ^*∗*^ *P* < 0.05 (24 h of LSS versus static).

Krüppel-like factor-2 (KLF-2) expression was studied, as KLF-2 is a transcriptional factor known to be induced by LSS [21, 22]. Interestingly, a significant decrease of KLF-2 mRNA expression was observed after 24 h of LSS, (0.37 ± 0.21) (Figure 3(a)). A significant increase of KLF-2 mRNA expression was also observed at 10 min (1 versus 16.78 ± 0.84) but not at 24 h. A significant decrease of KLF-2 mRNA expression was also observed after 24 h of LSS, compared to 10 min of LSS (16.8 ± 0.84 versus 1.64 ± 0.07) (Figure 3(a)).

### 3.4. Regulation of miR-126 Levels in Endothelial Cells under Static and LSS Conditions

To assess miR-126 changes after LSS treatment, miRNAs were quantified by qRT-PCR (Figure 3(b)). A significant decrease of miR-126 expression in HUVECs was demonstrated after 10 min of LSS, compared to control static conditions (1 versus 0.58 ± 0.13). A significant increase of miR-126 expression was also observed after 24 h of LSS, compared to 10 min of LSS (0.58 ± 0.13 versus 1.88 ± 0.29). Our data also demonstrated that 24 h of LSS significantly decreased SDF-1/CXCL12 by 98% (1 versus 0.12 ± 0.06). In order to assess the specificity of miR-126 response to LSS, we studied another miR from endothelial cells, miR-222 (data not shown) and found that it was unaffected by a 24 h flow.

### 3.5. Upregulation or Downregulation of miR-126 Does Not Affect HUVEC Proliferation or Apoptosis

**Figure 4:**
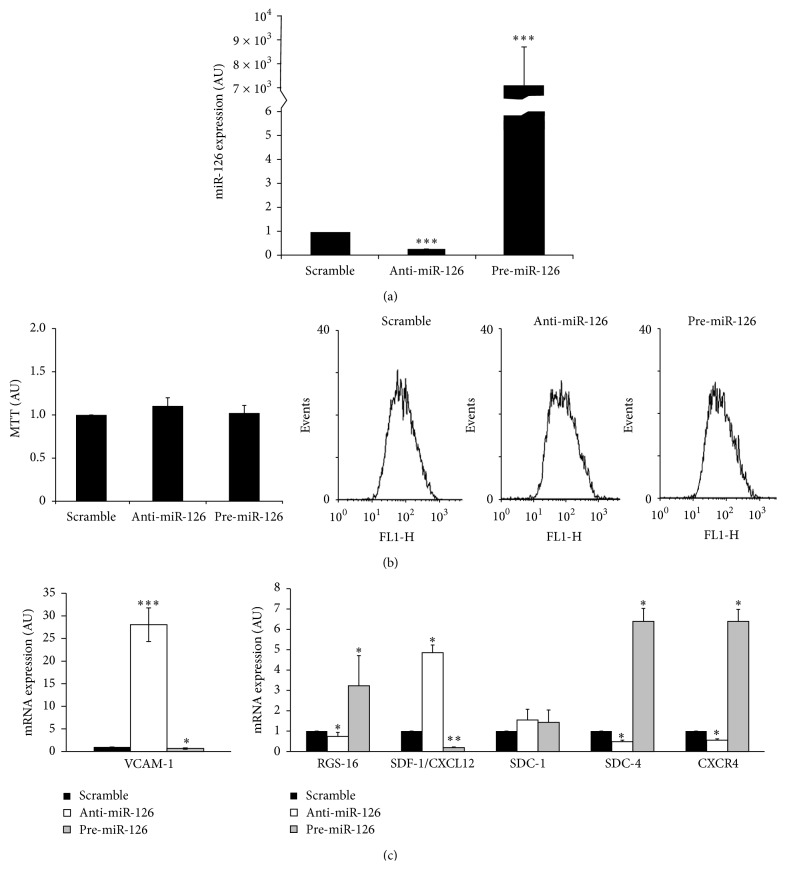
Study of HUVEC transfection after 48 h with scramble, anti-miR-126, and pre-miR-126 under static conditions. (a) Confirmation of miR-126 overexpression and knockdown under the respective conditions. Values are expressed as mean ± SD of 3 independent experiments (AU: arbitrary units). Student's *t*-test, for miR-126: ^*∗∗∗*^ *P* < 0.0001 (scramble versus anti-miR-126), ^*∗∗∗*^ *P* < 0.0001 (scramble versus pre-miR-126). (b) HUVEC metabolic activity was measured using MTT test after HUVEC transfection. Representative flow cytometry histogram study of apoptosis from HUVECs. (c) Expression of various mRNA targets in HUVECs after 48 h of transfection with anti-miR-126 and pre-miR-126. Values are expressed as mean ± SD of 3 independent experiments (AU: arbitrary units). Student's *t*-test, for VCAM-1: ^*∗∗∗*^ *P* < 0.0001 (scramble versus anti-miR-126), ^*∗*^ *P* < 0.05 (scramble versus pre-miR-126); for RGS-16, SDC-4, CXCR4: ^*∗*^ *P* < 0.05 (scramble versus anti-miR-126), ^*∗*^ *P* < 0.05 (scramble versus pre-miR-126); for CXCL12: ^*∗*^ *P* < 0.05 (scramble versus anti-miR-126), ^*∗∗*^ *P* < 0.001 (scramble versus pre-miR-126).

Pre-miR-126 and anti-miR-126 sequences were initially transfected into HUVECs and compared to the scramble control (Figure 4(a)). A marked decrease of miR-126 expression was observed after anti-miR-126 transfection (1 versus 0.23 ± 0.034) and a marked increase of miR-126 expression was observed after pre-miR-126 transfection (1 versus 7000 ± 1168), compared to controls (Figure 4(a)).

Secondly, the effect of anti-miR-126 or pre-miR-126 or scramble transfection on cell proliferation was analyzed by means of the MTT assay and apoptosis was studied by flow cytometry-Annexin V assays. No significant difference in terms of cell proliferation was observed between scramble, anti-miR-126, and pre-miR-126 transfected cells, compared to nontransfected control cells (Figure 4(b), left panel). No significant changes in HUVEC apoptosis measured by flow cytometry were observed after anti-miR-126 or pre-miR-126 or scramble transfection (Figure 4(b), right panels).

### 3.6. miR-126 Is a Negative Regulator for VCAM-1 and SDF-1/CXCL12 and a Positive Regulator for RGS16, CXCR4, and SDC-4 in HUVECs

Upregulation of miR-126 (transfection with pre-miR-126) increased RGS16 (1 versus 3.23 ± 1.48), SDC-4 (1 versus 6.39 ± 0.64), and CXCR4 expression (1 versus 6.39 ± 0.91) and decreased SDF-1/CXCL12 (1 versus 0.19 ± 0.03) and VCAM-1 expression (1 versus 0.65 ± 0.14) in HUVECs. However, no significant difference was observed for SDC-1 expression (1 versus 1.43 ± 0.61) after pre-miR-126 transfection (1 versus 0.87 ± 0.79) (Figure 4(c)).

In addition, miR-126 knockdown (transfection with anti-miR-126) decreased RGS16 (1 versus 0.74 ± 0.20), SDC-4 (1 versus 0.48 ± 0.08),  and CXCR4 expressions (1 versus 0.56 ± 0.23). A significant increase of SDF-1/CXCL12 (1 versus 4.8 ± 0.38) and VCAM-1 (1 versus 28.05 ± 3.7) expression was observed under the same conditions. No significant difference was observed for SDC-1 expression after anti-miR-126 transfection (Figure 4(c)).

### 3.7. miR-126 and SDC-4 Expression Increased in Apo-E KO/CKD Mice

**Figure 5:**
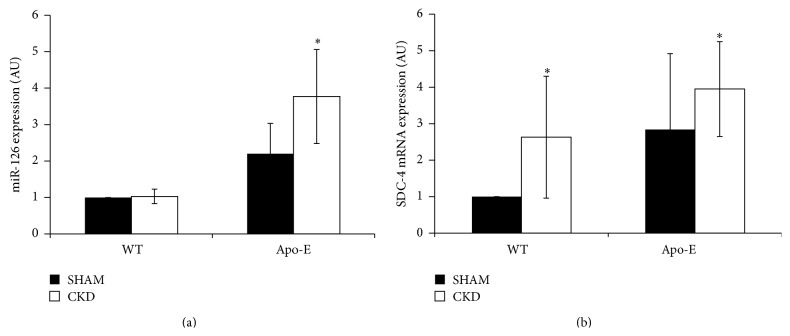
miR-126, miR-126, and SDC-4 expression in control and atherosclerotic mice. Twenty-week-old mice aortas were isolated from wild-type (WT) and Apo-E KO mice for miRNA and mRNA studies at the indicated times. CKD mice were subjected to 10 weeks of uremia. (a) miR-126 expression expressed as RQ normalized to U6. (b) SDC-4 expression normalized to GAPDH. Values are expressed as mean ± SD of 3 independent experiments (AU: arbitrary units). Student's *t*-test, for miR-126: ^*∗*^ *P* < 0.05 (WT sham versus Apo-E CKD); for SDC-4: ^*∗*^ *P* < 0.05 (WT sham versus WT CKD), ^*∗*^ *P* < 0.05 (WT sham versus Apo-E CKD).

To confirm our findings in an* in vivo* model, we decided to analyze SDC-4 expression in vessels from a rodent model where miR-126 was shown to be increased [16]. We thus measured concomitantly miR-126 and SDC-4 in aortas from mice models with CKD, atherosclerosis, and vascular calcification [16]. Wild-type (WT) C57/BL6 as well as Apo-E KO mice were submitted to partial nephrectomy to induce CKD. Apo-E KO mice characteristically develop large atheromatous plaques and low-grade vascular calcification and the combination of Apo-E KO/CKD leads to atherosclerosis and aortic calcification [16]. Experiments were performed 10 weeks after induction of uremia, when CKD mice presented severe uremia and Apo-E KO mice presented marked atherosclerosis (data not shown). Under these conditions, miR-126 and SDC-4 expression were also correlated* in vivo*, as miR-126 expression was increased in the aorta of Apo-E KO/CKD mice (1 versus 3.77 ± 1.29), compared to WT SHAM mice (Figure 5). A significant increase of SDC-4 expression was also observed in WT CKD (1 versus 2.63 ± 1.67) and Apo-E KO/CKD mice (1 versus 3.95 ± 1.3), compared to WT SHAM mice (Figure 5).

## 4. Discussion

LSS has been associated with a vasoprotective endothelial phenotype [5]. The present study demonstrates that LSS is associated with human endothelial cell (HUVEC) elongation after an intermediate exposure time to flow, but when submitted to longer exposure times to LSS, HUVECs changed their phenotype to a cobblestone shape with rearrangement of the cytoskeleton distribution in the cell, especially near to membrane, and increased proteoglycan expression. Increased shear stress induces tension which is accommodated by the endothelial cytoskeleton* via *rearrangement of actin and intermediate filament proteins at sites of cell-matrix contact [23]. When HUVECs were submitted to prolonged LSS, downregulation of adhesion molecule VCAM-1 was observed concomitantly with mechanical reorganization of the cytoskeleton. These changes were reflected by significant upregulation of miR-126 during long-term exposure to flow. These changes suggest that flow could confer an anti-inflammatory and atheroprotective phenotype to vascular endothelial cells.

It has been previously reported that mechanical and haemodynamic phenomena applied the forces to arterial walls and especially shear stresses to the vascular endothelium [24]. There are many values of shear in various vessels, <10 dyne/cm^2^ (for vena cava, large veins, descending aorta), <20 dyne/cm^2^ (for venules and ascending aorta), and <60 dyne/cm^2^ (for capillaries and arterioles) [25]. In our study, we used shear stress at 0.8 dyne/cm^2^, since this condition is optimal to study the mechanical properties of shear flow on adhesion molecule VCAM-1 and syndecans expression and to understand their role in blood-cell adhesion on endothelium [20].

VCAM-1 is expressed by vascular endothelial cells and plays an important role in monocyte or lymphocyte rolling and adhesion in early-onset atherosclerosis [26] and in the inflammatory process [27]. The decreased VCAM-1 expression observed in this study suggests that long-term LSS confers anti-inflammatory properties to endothelial cells, as already reported [22].

We therefore decided to analyze genes critical in conferring atheroprotective properties to the endothelial cell surface and investigated the expression of transcription factor KLF-2.

KLF-2is a zinc-finger transcription factor that is exclusively present in the adult vasculature and has distinct functions in vasomotor regulation, inflammation, homeostasis, and angiogenesis. In cardiovascular biology, KLF-2 is described to be an important molecular transducer that converts hemodynamic forces into the regulation of gene expression [28, 29]. Some studies have already reported results in favour of increased KLF-2 transcription when cells were subjected to laminar flow [28, 30, 31]. Shear stress induces KLF-2 by raising mRNA and protein levels by transcriptional activation at the KLF-2 promoter via the MEK5/ERK5 Mitogen-activated protein kinases (MAPK) cascade which leads to activation of myocyte enhancer binding factor 2 (MEF2) at the KLF2 promoter [21, 32]. In addition, van Thienen et al. [29] showed that shear stress induces KLF-2 mRNA stabilization.

In the present study, we observed an acute increase of KLF-2 mRNA after 10 min of exposure to LSS, followed by a decrease after 24 h. We therefore conclude that in our experimental condition LSS does not continuously sustain KLF-2 mRNA transcription and/or stabilization.

miRNAs have been described as important modulators of angiogenesis and atherosclerosis [33]. We measured miR-126 expression, as it is the most abundant miRNA found during endothelial cell (EC) differentiation and in adult ECs [12] and it is deregulated in various cardiovascular disorders [14]. miR-126 was diversely regulated when exposed to LSS. After a brief exposure to LSS (10 min), a slight decrease of miR-126 expression was observed concomitant with an increase of KLF-2 mRNA and, after a long exposure time to LSS (24 h), a marked increase of miR-126 expression was observed. This increase could possibly be induced by the increased KLF-2 expression observed after 10 min of exposure, as KLF-2 has been shown to increase miR-126 expression in zebrafish [31]. But Harris et al. [34] found a potential binding site for KLF-2 in the Egfl7/miR-126 5′ flanking region. In comparison, Hergenreider et al. used on HUVECs a higher shear at 20 dyne/cm^2^ for a longer time (3 days) and found no effect on miR-126 expression level and no significant regulation of miR-126 by KLF-2 overexpression [30]. On the other hand, Schober et al., 2014, showed that shear stress on HUVECs induces KLF-2-dependent expression of pri-miR-126 but not miR-126-3p [35].

miR-126 is known to regulate many targets either directly (SDF-1/CXCL12, CXCR-4, VCAM-1 and RGS16) and/or indirectly (SDF-1/CXCL12, CXCR-4) [12, 13, 17, 36]. We did not find any evidence that SDC-4 is a direct target of miR-126 by looking at all known dedicated databases. Our results show that the increase in miR-126 after 24 h of LSS was concomitant with downregulation of its well-known target VCAM-1 at the protein level compared to 10 min LSS and miR-126 overexpression experiments resulted also in a decrease of VCAM-1 mRNA. In the same way, Harris et al. [12] observed that overexpression of miR-126 decreases VCAM-1 protein expression and consequently reduces leukocyte adhesion to endothelial cells. The changes we observed after 10 minutes of flux are probably not consequent to a change in gene expression but are likely due to other mechanisms such as the last steps of protein biosynthesis or phosphorylation of kinases.

As expected, overexpression of miR-126 under our experimental conditions provided results comparable to those published by van Solingen et al. [14], that is, a decrease of SDF-1/CXCL12 and VCAM-1, which are described targets of miR-126 [12]. This effect occurred in the absence of apoptosis. In contrast, Zernecke et al. showed that the increase in miR-126 expression was concomitant with enhancement of SDF-1/CXCL12 in an indirect pathway mediated by apoptosis [17]. Knowing that miR-126 decreases SDF-1/CXCL12 and VCAM-1 expression, we can hypothesize that overexpression of miR-126 is atheroprotective and may lead to a decrease of leukocyte homing from the blood circulation through the endothelium* in vivo* [29, 37]. On the other hand, miR-126 modulation (under- or overexpression) did not change in our hands cell proliferation or apoptosis. Van Solingen et al. showed in a mouse hindlimb ischemia model that modulating miR-126 does not affect shear-stress-induced arteriogenesis [38]. The same authors found also that short-term antagomir-126 treatment did not alter HUVEC cell migration or proliferation which is concordant with our present results.

It has been clearly documented that the remodelling of endothelial cells due to LSS is associated with an increase of proteoglycan distribution and marked modification of gene expression, resulting in protection of the endothelial layer from atherosclerosis [8, 9]. We therefore decided to investigate the link between the SDF-1/CXCL12/SDC-4 complex and miR-126, as SDF-1/CXCL12, another target of miR-126, is known to form a stable complex by binding SDC-4, which in turn binds CXCR4 [14, 17, 39]. We have previously shown that SDC-4 behaves like a SDF-1/CXCL12 coreceptor. In this study we explored the effect of upregulation of miR-126 on SDF-1/CXCL12, CXCR4, and SDC-4 expression and their impact on actin cytoskeleton remodeling. Interestingly, a new finding of our study is that miR-126 is a positive regulator of SDC-4, as miR-126 overexpression increased the level of SDC-4 mRNA. miR-126 overexpression was a positive regulator of CXCR4 in our model. This result is discordant with those of a study in colon cancer cells, which demonstrated that miR-126 is a negative regulator of CXCR4 expression [40]. We suggest that our model comprises a more complex system because transfection of miR-126 significantly decreased the SDF-1/CXCL12 level and increased its receptor CXCR4.

We decided to study whether our* in vitro* findings were mirrored in an* in vivo* model where miR-126 is increased. As expected, we confirm here that miR-126 expression is significantly increased in aorta from Apo-E KO mice with CKD as previously described [16]. We show here that, in mice expressing a high level of miR-126 in their aortas (Apo-E KO/CKD mice), the SDC-4 was also significantly increased. Apo-E KO/CKD mice present higher calcification and atherosclerosis levels than WT CKD mice; this could explain why we did not find an increase in WT CKD mice for miR-126 expression. Taken together, our* in vivo* findings therefore mirror our in vitro results, suggesting that miR-126 and SDC-4 levels are concomitantly upregulated in vivo in ApoE/CKD mice aortas and in vitro in endothelial cells [16].

**Figure 6:**
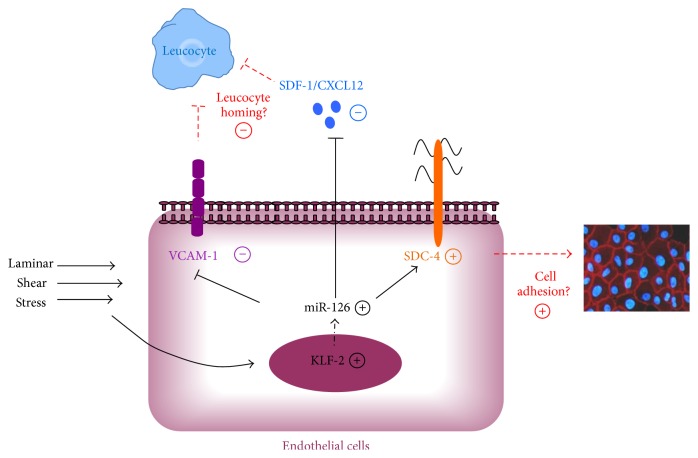
Representative network of miR-126 in HUVECs submitted to LSS. LSS induces at short-term the expression of transcription factor, KLF-2, and at long-term miR-126 expression. miR-126 downregulates the endothelial adhesion molecule VCAM-1 and chemokine SDF-1/CXCL12. Loss of VCAM-1 and SDF-1/CXCL12 can be associated with decrease of leukocyte homing over the endothelium. miR-126 overexpression enhanced the SDC-4 which can induce the transduction pathway leading to remodeling of F-actin cytoskeleton and favors cell adhesion and spreading. VCAM-1: vascular cell adhesion molecule-1; miR-126: small noncoding micro-RNA-126; SDF-1/CXCL12: stromal cell-derived factor-1; KLF-2: Krüppel-like factor-2; SDC-4: syndecan-4.

In summary, our data demonstrate that LSS has an important impact on miR-126 expression in HUVECs (Figure 6), and its protein targets SDF-1/CXCL12. The results of our* in vitro* model confirm the previously described impacts of miR-126 on normal and inflammatory events. This study also highlights the potential role of miR-126 on regulation of syndecan-4 expression. Our finding emphasizes the importance of identifying different mechanisms to explain this regulation.
